# PD20 Evaluation Of Diagnostic Methods For Visceral Leishmaniasis Detection: A Systematic Review

**DOI:** 10.1017/S0266462325101554

**Published:** 2025-12-29

**Authors:** Mariana del Grossi, Bruna Santos, Stéfani Borges, Daniela Melo

## Abstract

**Introduction:**

Visceral leishmaniasis (VL), caused by *Leishmania infantum*, is a significant public health challenge in the Americas and the Mediterranean basin. Effective disease control requires timely and accurate diagnostic methods, particularly in endemic regions. This study systematically evaluated the diagnostic accuracy of polymerase chain reaction (PCR), the rK39 rapid test, and ELISA with respect to their sensitivity and specificity against parasitological examination across diverse populations.

**Methods:**

A systematic review was conducted using MEDLINE, Embase, the Cochrane Library, and LILACS databases without restrictions on publication date, language, or whether the study was published, unpublished, or ongoing. Studies assessing the diagnostic accuracy of PCR, the rK39 rapid test, and ELISA for detecting VL against direct parasitological examination were included based on predefined PIROS framework (Population, Index test, Reference standard, Outcomes, and Study design). Study selection and data extraction were independently performed by two reviewers, with disagreements resolved by a third reviewer. Data were synthesized narratively and through meta-analyses to estimate sensitivity and specificity. Methodological quality was assessed using QUADAS-2 and evidence certainty was evaluated using the GRADE approach.

**Results:**

Twenty studies were included. In immunocompetent patients, PCR had high sensitivity (91 to 98.5%) and specificity (up to 100%) for detecting VL, providing a less invasive alternative to bone marrow aspiration. The rK39 test had sensitivity of 82.4 to 97 percent and a specificity close to 100 percent, which is suitable for endemic regions. ELISA sensitivity ranged from 89 to 94 percent. In immunosuppressed patients, PCR retained superior accuracy (83 to 100% sensitivity), while the rK39 sensitivity declined to 60 to 67 percent. Certainty of the evidence was moderate for immunocompetent patients but low for immunosuppressed groups, due to heterogeneity and the limited number of studies.

**Conclusions:**

PCR was the most accurate method for detecting *Leishmania infantum* in endemic regions, especially for immunosuppressed patients. The rK39 rapid test was effective for initial screening in high-prevalence areas, and ELISA was helpful in specific contexts. These results emphasize the need for non-invasive, accurate diagnostics. Future studies should address evidence limitations, particularly for vulnerable populations.

